# Analysis of Monomer Release from Different Composite Resins after Bleaching by HPLC

**DOI:** 10.3390/life12111713

**Published:** 2022-10-27

**Authors:** Mehmet Gökberkkaan Demirel, Hakan Yasin Gönder, Makbule Tuğba Tunçdemir

**Affiliations:** 1 Department of Prosthodontics, Faculty of Dentistry, Necmettin Erbakan University, Yaka Mah, Bağlarbaşı Sok, No.4, 42090 Meram, Konya, Turkey; 2 Department of Restorative Dentistry, Faculty of Dentistry, Necmettin Erbakan University, Yaka Mah, Bağlarbaşı Sok, No.4, 42090 Meram, Konya, Turkey

**Keywords:** bleaching applications, bulk-fill resin composites, high-performance liquid chromatography, monomer release, nanohybrid resin composites

## Abstract

(1) Background: This study aimed to examine the effect of bleaching agents on the release of triethylenae glycol dimethacrylate, 2-hydroxyethyl methacrylate, bisphenol A, urethane dimethacrylate, and bisphenol A-glycidyl methacrylate monomers, which are released from different composite resins, using the high-performance liquid chromatography (HPLC) method. (2) Methods: Ninety disc-shaped specimens were produced and immersed in artificial saliva. After different bleaching applications [office type bleaching (OB) and home type bleaching (HB)], the specimens were immersed in a 75 wt% ethanol/water solution, and the released monomers were analyzed by HPLC at predefined time intervals: 1, 7, and 28 days. The Kruskal–Wallis and Mann–Whitney U tests were conducted for statistical analysis (*p* = 0.05). (3) Results: The monomers were released at all times from all composite specimens. The monomer release was increased over time. The highest monomer release was detected on day 28. Bleaching applications affected monomer release. No statistical difference was found between OB and HB applications (*p* > 0.05). The most released monomer was Bisphenol-A in all composites. (4) Conclusion: Given that a residual monomer release from composite resins has a toxic effect and that bleaching treatments increase this release, a treatment protocol should be made in accordance with the manufacturer’s instructions.

## 1. Introduction

Resin-based composites (RBC) are commonly used because they can be applied easily, enable conservative work, and provide an aesthetically pleasing appearance. Resin-based composites are essentially composed of an organic resin matrix, an inorganic filler, and a silane agent that bonds the two parts together [1].

The most commonly used monomers in a resin matrix are bisphenol A-glycidyl methacrylate (Bis-GMA), triethylene glycol dimethacrylate (TEGDMA), urethane dimethacrylate (UDMA), and bisphenol A (BPA) [2]. Previous studies have shown that RBC content is degraded in the oral environment [3,4]. This places doubt on the biocompatibility of the material. Researchers have reported that the dimensions of monomers, the number of monomers with incomplete polymerization, and the oral environment’s ability to degrade are effective factors in degradation [5].

The toxic effects of monomers released from resin-based composites have been investigated, and Bis-GMA, TEGDMA, and UDMA monomers have been reported to have high toxicity potential [6,7]. Researchers have defined unreacted TEGDMA, Bis-GMA, and UDMA as toxic substances with cytotoxic, genotoxic, mutagenic, and allergic effects [6,8]. Moreover, they have been reported to increase the growth of cariogenic microorganisms that colonize marginal space [9]. Originally developed as a synthetic estrogen, BPA is an endocrine-disrupting compound. It interacts similarly with natural hormones and blocks the normal action of hormone receptors [6]. It has been reported that incomplete polymerization, or the disintegration of resin over time, leads to the degradation of BPA [10].

The amount of conversion of monomers to polymers may vary depending on endogenic factors, such as the chemical structure of monomers, the concentration of catalysts, and exogenous factors, such as polymerization conditions [11]. A sufficient intensity of light in resin-based composites is important to achieve adequate polymerization, and this depth is suggested to be 2 mm in conventional resin-based composites [12]. Applying an incremental technique is necessary for applying resin-based composites in deeper cavities. In this technique, the space between layers, the risk of contamination, the difficulty in manipulation due to limited access in small cavities, and the prolonged treatment time [13] of this technique pushed researchers to search anew for other techniques, and thus, bulk-fill composites (BF) were developed.

BF is a newly designed resin-based composite derivative that allows placement in the cavity in larger masses (4 mm). Manufacturers developed this using more translucent fillers and photoinitiators. Although it is considered that the number of unpolymerized monomers can be reduced by using this material, its conversion amount is lower than that of conventional resin-based composites [14].

Nanohybrid (NH) composites, a recently developed resin-based composite derivative, contain macro- and micro-sized particles and nano-sized filling materials. The nano-scale fillers provide fewer spaces in the mass by filling the spaces in the mass. These nano-fillers have a smaller particle size than the wavelength of light, and NHs are produced to combine the mechanical properties of macrofilled composites with the optical properties of microfilled composites [15].

Dental bleaching treatments have become very popular today due to patients’ high aesthetic expectations. Applied in two ways as at-home and in-office, bleaching agents are applied to patients together with different forms and concentrations of hydrogen peroxide (HP) or carbamide peroxide (CP) in their structure. [16] When CP is exposed to saliva during the bleaching procedure, it reacts and produces HP and urea. Free radicals released after this chemical reaction may increase monomer release by accelerating the hydrolytic degradation of resin composites or by affecting the filler-matrix link [17].

It is necessary to understand the degradation mechanism of resin-based materials to determine the type and number of monomers released and their importance in clinical use. Therefore, in this study, monomer release was investigated by applying two different types of bleaching agents to three different types of composites to examine the effect of bleaching agents on different composites in terms of monomer release; the elution of monomers was then determined using high-performance liquid chromatography (HPLC). In previous studies, the HPLC method was used to investigate the eluted monomers. [18,19] Although previous studies have compared NHs among themselves, [20] or with conventional resin composites, [18,21,22,23] and BFs among themselves, [4,24,25] or with conventional resin composites, [19] with or without applying bleaching agents. No study has yet compared BFs with NHs or three of them with each other.

This study aimed to compare the amount and type of monomer release by applying bleaching agents to commonly used BFs, NHs, and suprananohybrid (SN) resin composites. The initial hypothesis of the study is that as a result of applying different bleaching agents to different resin composites, the monomer release will be similar and will not change over time.

## 2. Materials and Methods

Table 1 shows the composite types and bleaching agents used in the study, and Table 2 shows the monomers.

### 2.1. Preparation of Specimens

The specimens were prepared (30 specimens from each composite) using a cylindrical plastic mold (diameter: 8 mm, thickness: 4 mm). The lower surface of the slots in the mold was fixed with an adjustable plastic bar, the cavity was filled with an appropriate amount of resin composite, and pressure was applied to its upper surface using glass to obtain a smooth surface. The specimens were polymerized following the manufacturer’s instructions as follows: BF specimens, 30 s, one layer applied (layer thickness: 4 mm); NH specimens, 20 s, two layers applied (layer thickness: 2 mm); and SN specimens, 10 s, two layers applied (layer thickness: 2 mm). For the polymerization of all specimens, a LED unit (Elipar S10, 3M, St. Paul, MN, USA) with 1200 mW/cm^2^ light intensity was used.

The specimens were polished with a water-cooled polishing kit (Enhance PoGo Complete Kit, Dentsply Sirona, NC, USA) at low speed, and each specimen was placed in 5 mL of artificial saliva in an amber sample bottle. The artificial saliva contained 4.1 mM potassium dihydrogen phosphate, 4.0 mM disodium hydrogen phosphate, 24.8 mM potassium bicarbonate, 16.5 mM sodium chloride, 0.25 mM magnesium chloride, 4.1 mM citric acid, and 2.5 mM calcium chloride [20]. For the specimens to simulate the oral environment, they were kept in artificial saliva for 72 h at room temperature.

### 2.2. Bleaching Application

Two different bleaching agents were used: an in-office (OB) chemically activated bleaching agent (Opalescence Boost) and an in-home (HB) chemically activated (Opalescence PF) bleaching agent.

The composite specimens were randomly divided into three subgroups: HB group (HBG), OB group (OBG), and control group (CG) (*n* = 10). OBG was applied for 45 min in total (3 × 15 min) and HBG for 56 h (4 h × 14 days) to the specimens kept in artificial saliva for 72 h. In the non-bleaching period, the specimens were immersed again in artificial saliva. CG continued to be immersed in saliva throughout this process. All procedures were meticulously performed by a single experienced physician.

### 2.3. Storing Specimens in Solvent

Following the final bleaching procedure, the specimens were washed with deionized water using a soft brush, and then 10 samples of each material were randomly immersed into a test tube in 1 mL of 75% ethanol solution. The mouth of the test tube was sealed using parafilm grafting tape. At the end of days 1, 7, and 28, the solution was placed into a vial tube using a syringe filter and transferred to the laboratory for HPLC analysis.

### 2.4. HPLC Monomer Analysis

TEGDMA, Bis-GMA, bisphenol A, UDMA, and HEMA monomers were investigated by HPLC. HPLC was performed on an isocratic HPLC instrument (Shimadzu LC-20AT Prominence) with an LC-20AT pump, a manual injector with a loop volume of 20 µL, and a programmable variable wavelength Shimadzu SPD-20A detector. Separation was carried out with a Supelcosil LC-18 reverse-phase column (4.6 × 250 mm, 5 µm particle size). The mobile phase was 65% acetonitrile (gradient grade, Sigma-Aldrich, St. Louis, MO, USA) and 35% water (Direct-Q 3 UV system, Millipore, Burlington, MA, USA). The flow rate of the mobile phase and the run time were set to 1 mL/min and 12 min, respectively. The column temperature was 25 °C. The detection wavelength was 205 nm, and the chromatograms were analyzed using LC Solution (Shimadzu, Kyoto, Japan) software.

The qualitative and quantitative evaluations of monomer release were carried out according to the report of Pelka et al. [26]. A mixture of 10 mg of each of the monomers (Table 2) in a 4 mL acetonitrile/water mixture (1:1) was used as standard. Standard HPLC spectra were obtained by injecting this mixture into the device at appropriate rates. The retention times and peak values of the monomers were recorded, and the concentrations were determined in μmol/L based on the calculation of the area under the peaks obtained from the standard solutions.

### 2.5. Statistical Analysis

Statistical package software (SPSS 21, IBM, Chicago, IL, USA) was used to analyze the data. The Kolmogorov–Smirnov test was used to determine the normality of the data. Nonparametric tests were performed because the variances of the groups were not homogeneous.

The Kruskal–Wallis and Mann–Whitney U tests were used to determine whether a significant difference existed between the time periods, monomers, and bleaching applications in terms of the number of monomers released from the composite resins. A *p* value < 0.05 was considered statistically significant.

## 3. Results

**Figure 1:**
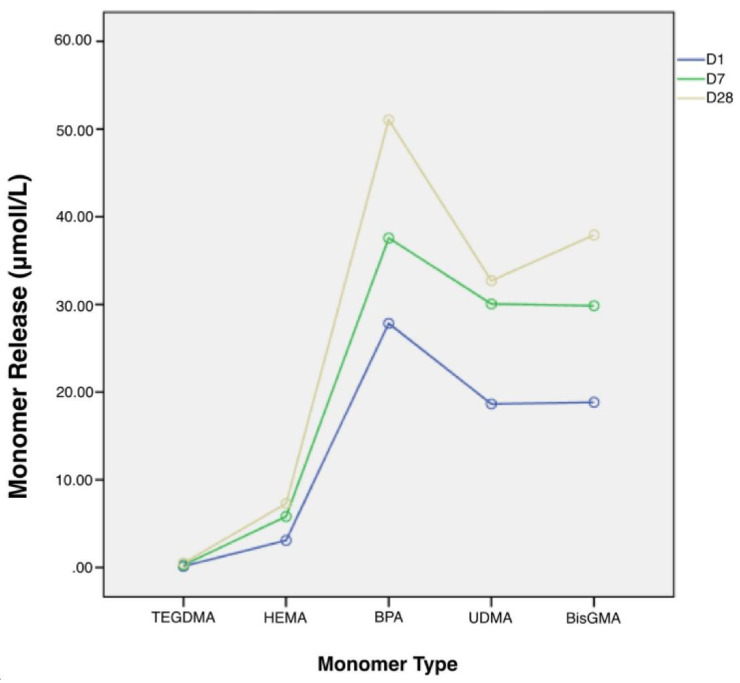
Total Monomer Release.

The monomers were released at all times from all composite specimens. According to the Kruskal–Wallis test results, a statistically significant difference was found between composite resins, monomers released, and measurement days (*p* < 0.05) (Figure 1).

Compared with other composites, monomer release was significantly high in the SN specimens. Among all composites, monomer release was found to be the least in the BF specimens. In terms of measurement days, monomer release was the highest on day 28, and it was observed to increase over time (Table 3).

A statistically significant difference was found in the evaluation of the five different monomers (*p* < 0.05) (Table 3). Monomer release was the highest in BPA, followed by Bis-GMA, UDMA, and HEMA. Monomer release was the least in TEDGMA. According to the pairwise comparison, whereas no significant difference was found between BPA and UDMA, a statistically significant difference was observed between the other monomers (*p* < 0.05) (Table 1). No significant difference was found between the different types of bleaching agents and the control group (*p* > 0.05) (Table 3). Table 4, Table 5, Table 6, Table 7 and Table 8 show the monomer types and the amounts released after the application of the two bleaching products to the composite resins after 1, 7, and 28 days, respectively.

TEGDMA release increased both over time and with bleaching applications for all composite types. Although the time-dependent increase between day 7 and day 28 was not significant in general, it was significantly higher than that on day 1 overall. In terms of bleaching applications, although no significant change was observed between HB and OB in general, these groups caused significantly more monomer release than group C. The order of release between the composites is as follows: BF > NH > SN (Table 4).

HEMA release also increased both over time and with bleaching applications for all composite types. Although the time-dependent increase between day 7 and day 28 was not significant in general, it was generally significantly higher than that on day 1 for these values. In terms of bleaching applications, although no significant change was observed between HB and C in general, these groups caused significantly less monomer release than OB. The order of release between the composites is as follows: SN > NH > BF (Table 5).

BPA release increased significantly over time for all composite types. Bleaching applications did not cause a significant change in terms of BF in general. Although no significant change was observed between HB and OB for NH and SN, they caused significantly more monomer release than group C. The order of release between the composites is as follows: SN > BF > NH (Table 6).

UDMA release increased over time for all composite types, but the difference between day 7 and day 28 was not significant. Bleaching applications did not cause a significant change for BF and NH in general but caused a significant increase for SN (Table 7).

Bis-GMA release increased over time for all composite types, but the difference between day 7 and day 28 was not significant. Bleaching agent application did not cause a significant change in the overall composite resins (Table 8).

## 4. Discussion

In the present study, different bleaching agents were applied to three different resin composites, and the release amounts of Bis-GMA, TEGDMA, UDMA, BPA, and HEMA were evaluated time-dependently. The hypothesis stating that the monomer release of different resin composites after applying different bleaching agents would be similar was accepted, and the hypothesis suggesting that the “release would not change over time” was rejected.

Materials used in dentistry are expected to be biocompatible and not to show any toxic, allergic, or harmful effects on surrounding tissues. Previous studies have revealed that bleaching applications may increase water absorption by increasing the surface roughness and decreasing the surface hardness of resin composites, and thus, they may lead to a loss of filler [17,27]. However, the findings of this study showed that although bleaching applications increased monomer release to a certain extent, this increase was not statistically significant in every situation. Durner et al. reported that the bleaching application increased the monomer release, but they attributed this result to the fact that after the bleaching application, they wiped the bleaching agent off with cotton rather than washing it [17]. In fact, researchers who washed off the bleaching agents from the specimens after the bleaching application reported that the application did not significantly affect the monomer release [18,20,21]. The reason why the monomer release was not significantly affected by the bleaching application in this study may be due to the bleaching agents affecting the resin composite specimens only for a limited time and being washed away.

The cytotoxicity and genotoxicity of monomers released from resin composites have been investigated, and their effect on cellular functions, such as cell proliferation and viability, inhibition of enzyme activity, or membrane integrity, has been studied. Issa et al. found that released monomers reduce mitochondrial activity, [28] and Lefeuvre et al. reported that TEGDMA causes mitochondrial damage [29]. Moreover, HEMA, TEGDMA, and UDMA have been reported to inhibit the cell growth of gingival fibroblasts by depleting the presence of glutathione, a very important antioxidant for cellular functions. [30] Previous studies have revealed that BPA is an endocrine-disrupting compound that inhibits the continuity of meiotic division and leads to changes in the cell cycle [31]. Engelmann et al. reported that Bis-GMA causes a rapid and intense decrease in gingival fibroblasts compared with TEGDMA even at low concentrations [32].

Toxic concentrations of these monomers have been reported in previous studies. Reichl et al. found that the toxic concentrations of TEGDMA, UDMA, and Bis-GMA affecting oral mucosa cells were 3700, 270, and 110 μmol/L, respectively [33]. Kita et al. suggested that the concentration values of BPA above 10 μmol/L showed estrogen-like effects [34]. According to Cataldi et al., a HEMA dose above 3000 μmol/L would have toxic effects on gingival fibroblasts [35].

Similar to the literature, [20,21,23,24] the lowest monomer release in this study was observed for TEGDMA in all composite types. The low molecular weight and viscosity and the high reactivity of TEGDMA increased the cross-linking potential and the degree of conversion [36]. This might have caused the proportion of unreacted TEGDMA monomer to be too low in the matrix and have led to the low TEGDMA release. Moreover, as shown in this study, the concentration of TEGDMA monomer released from all resin composite specimens was below the toxic limits.

HEMA is a co-monomer that is added to the resin matrix because of its hydrophilic properties [25]. None of the manufacturers’ data on resin composites used in this study showed HEMA as a monomer present in the composition, as it was found to be released from all resin composite specimens at certain amounts. This could be attributed to the HEMA produced by the decomposition from UDMA. [4,24] The HEMA release identified in this study was low for the BF and NH specimens and far below the toxic limits. Although a statistically higher rate of HEMA release was found in the SN specimens, the amount released was again below the toxic limits. This may be due to HEMA not being directly present in the composition of resin composites and HEMA emerging at low rates as a side product of UDMA.

Although dental resin composites do not contain BPA, their structures usually contain monomers, such as Bis-GMA derived from BPA, bisphenol A dimethacrylate, bisphenol A polyethylene glycol dimethacrylate, 2,2-bis-[4-(3methacryloxy propoxy) phenyl] propane, and ethoxylated bisphenol A dimethacrylate [21,37,38]. In previous studies, BPA was observed in the aqueous medium, even though it was not found in the resin composites used [20,21,37]. This is probably due to the fact that it emerged as an impurity product that could have occurred during the synthesis process and/or as a degradation product of BPA-based monomers [37]. In this study, although the rate of BPA released from NH specimens was below the toxic limit, it was approximately within the toxic limit in the BF specimens and above the toxic limit in the SN specimens.

UDMA is added to the structure of resin composites to improve their mechanical properties and provide an alternative to BPA-based dimethacrylates. Owing to the absence of hydroxyl groups in its structure, it contributes to the reduction of water absorption [39]. The amount of UDMA released in this study was NH, BF, and SN, respectively. According to the manufacturer’s data, the composition of NH and BF contains UDMA, and the reason for the higher UDMA release from these specimens may be that they contain more UDMA monomers in their structure. Moreover, the most released monomer from NH specimens was UDMA. This result supports studies that previously examined monomer release from NH [21,23]. Although UDMA was highly released from the NH and BF specimens, the UDMA concentration released from all specimens was found to be below the toxic dose.

Bis-GMA is a monomer usually added to the structure of resin composites. It reduces polymerization shrinkage, increases mechanical properties, and provides a high level of bonding to enamel [40]. However, it has a low degree of conversion and high viscosity. [36] This study determined that Bis-GMA is generally released at a high rate, similar to previous studies [23,24,41]. This may be due to the generally low conversion of the double bonds of Bis-GMA [39,41] or to the fact that it shows a continuous and significant release, as it is a hydrophobic monomer with similar solubility parameters to ethanol, a non-polar organic solution [4,41].

Examining the cumulative sum of monomers released, monomer release was found to be SN > NH > BF. This is probably due to the difference in light cure times recommended by the manufacturers of resin composites. Increased curing time results in a higher level of polymerization and a decrease in the amount of monomer released [42,43]. Although it was not an objective of this study, a reduced monomer release as a result of increasing curing time was observed in the findings. Nevertheless, the number of monomers released from SN specimens was too high to be explained by the curing process. Another reason for this may be that aside from the short curing time, the titanium dioxide contained in its structure reacts with the hydroxyl groups and creates more severe surface alterations and the disrupted polymer networks cause more monomer release [18].

The gas chromatography technique can separate high-molecular-weight monomers, such as Bis-GMA and UDMA, into their molecules, causing only the dissociated molecules to be detected [24]. The HPLC technique decomposes the non-polar components of resin composites by their hydrophobic properties and enables the analysis of the decomposition process in a controlled manner by enabling the monomers to solute in the mobile phase. [3] For this reason, HPLC analysis, a powerful separation technique for examining monomers released from dental resin composites, was used frequently in previous studies [4,18,19,20,23,24].

Teeth and dental restorations may be exposed to different environments, from neutral to more acidic. Therefore, different solvents, such as acetonitrile, ethanol, methanol, artificial saliva, and distilled water, are used to examine monomer release. Polymerized resin composites consist of polymer networks containing unreacted monomers. By penetrating these polymer networks, the solvent expands the existing spaces and leads to the elution of unreacted monomers. [44] Previous studies have shown that solvents affect monomer release differently. As an aggressive solvent, ethanol enables the worst-case scenario to be predicted for the amount of monomer released from composite restorations [22]. Moreover, as solvents are clinically appropriate food/oral simulating fluids, the U.S. Food and Drug Administration recommends a 75% ethanol solution [18].

As the surface area of the composite material in contact with the aqueous medium was greater than that in the mouth in this in vitro study, the amount of monomer released did not exactly reflect the clinical picture. This can be regarded as a limitation of this study. The application of specimens to cavities prepared for natural teeth instead of preparing them as a disc can give more accurate results in future studies.

## 5. Conclusions

The following results can be drawn within the limitations of the study:

(1) Monomer release increases over time from resin composites.
(2) The application of bleaching agents is likely to increase monomer release.
(3) To reduce the residual monomer release, the polymerization time and application protocol should be followed according to the manufacturer’s instructions.

## Figures and Tables

**Table 1:** Materials used in the study.

Materiel	Type	Content	Producer
Filtek™ Bulk Fill Posterior	BF	AUDMA, UDMA, 12-dodecane-DMA 20 nm silica fillers, 4–11 nm zirconia fillers, YbF3 (100 nm) 58,% volume, 76,5% weight	3M ESPE
Tetric N-Ceram	NH	19–20% Bis-GMA, UDMA Barium glass, YbF_3_ (0.04–3 mm), 55–57% volume, 80% weight	Ivoclar Vivadent
Estelite Σ Quick	SN	Bis-GMA, TEGDMA silica-zirconia filler, composite filler 71% volume, 82% weight	Tokuyama Dental
Opalescence Boost	OT	40% H_2_O_2_	Ultradent
Opalscence PF	HT	16% CH_4_N_2_O·H_2_O_2_	Ultradent

BF; Bulk-Fill Composite, NH; Nanohybrid Composite, SN; Supra Nanohybrid Composite, HT; Chemically activated home-type bleaching agent, OT; Chemically activated office-type bleaching agent.

**Table 2:** Monomers evaluated in the study.

Monomers	Name	Chemical Formula	Molecular Weight	CAS Number
Bis-GMA	Bisphenol A glycidyl methacrylate	C_29_H_36_O_8_	513.00	1565-94-2
TEGDMA	Triethylene glycol dimethacrylate	C1_4_H_22_O_6_	286.32	109-16-0
UDMA	Urethane dimethacrylate	C_23_H_38_N_2_O_8_	470.56	41137-60-4
BPA	Bisphenol A	C_15_H_16_O_2_	228.29	80-05-7
HEMA	2-hydroxyethyl methacrylate	C_6_H_10_O_3_	130.14	868-77-9

**Table 3:** Mean values and standard deviations of cumulative monomer elution from different variations (μmol/L).

		n	Mean ± SD
Composite	BF	450	5.36 ± 0.24 ^A^
	NH	450	18.94 ± 1.29 ^B^
	SN	450	36.04 ± 1.99 ^C^
Day	D1	450	13.72 ± 1.0 ^A^
	D7	450	20.72 ± 1.45 ^B^
	D28	450	25.9 ± 1.86 ^C^
Monomer	TEGDMA	270	0.31 ± 0.02 ^A^
	HEMA	270	5.41 ± 0.48 ^B^
	BPA	270	38.83 ± 2.95 ^C^
	UDMA	270	27.14 ± 1.86 ^D^
	BISGMA	270	28.87 ± 1.53 ^D^
Bleaching	C	450	18.55 ± 1.4 ^A^
	HT	450	20.66 ± 1.53 ^B^
	OT	450	21.13 ± 1.55 ^B^

*p* < 0.05, D1; Day 1, D7; Day 7, D28; Day 28, BF; Bulk-Fill Composite, NH; Nanohybrid Composite, SN; Suprananohybrid Composite, C; No Bleaching Agent Application, HT; Home Type Bleaching Agent Application, OT; Office Type Bleaching Agent Application, Different superscript letters indicated a significant difference in groups.

**Table 4:** Mean values and standard deviations (Mean ± SD) of residual TEGDMA eluted from composites in different periods of time.

		D1	D7	D28
BF	C	^a^ 0.16 ± 0.03 ^A^	^d^ 0.21 ± 0.09 ^A^	^g^ 0.72 ± 0.21 ^B^
	HB	^b^ 0.24 ± 0.02 ^C^	^e^ 0.57 ± 0.28 ^D^	^g^ 0.82 ± 0.27 ^D^
	OB	^c^ 0.26 ± 0.02 ^E^	^f^ 0.65 ± 0.11 ^F^	^g^ 0.87 ± 0.26 ^F^
NH	C	^a^ 0.07 ± 0.0 ^A^	^d^ 0.14 ± 0.02 ^B^	^f^ 0.38 ± 0.06 ^C^
	HB	^b^ 0.09 ± 0.01 ^D^	^e^ 0.28 ± 0.05 ^E^	^fg^ 0.44 ± 0.04 ^F^
	OB	^c^ 0.11 ± 0.02 ^G^	^e^ 0.24 ± 0.05 ^H^	^g^ 0.47 ± 0.06 ^I^
SN	C	^a^ 0.05 ± 0.0 ^A^	^c^ 0.12 ± 0.01 ^B^	^e^ 0.18 ± 0.03 ^B^
	HB	^ab^ 0.08 ± 0.04 ^C^	^d^ 0.14 ± 0.01 ^C^	^f^ 0.28 ± 0.05 ^D^
	OB	^b^ 0.13 ± 0.01 ^E^	^d^ 0.16 ± 0.01 ^E^	^e^ 0.21 ± 0.02 ^F^

*n* = 10, *p* < 0.05, D1; Day 1, D7; Day 7, D28; Day 28, BF; Bulk-Fill Composite, NH; Nanohybrid Composite, SN; Suprananohybrid Composite, C; No Bleaching Agent Application, HB; Home Type Bleaching Agent Application, OB; Office Type Bleaching Agent Application, Different superscript lower case letters indicate the difference within the same column, and different upper-case letters indicate the difference within the same line.

**Table 5:** Mean values and standard deviations (Mean ± SD) of residual HEMA eluted from composites in different periods of time.

		D1	D7	D28
BF	C	^a^ 0.02 ± 0 ^A^	^c^ 0.05 ± 0.01 ^B^	^e^ 1.34 ± 4.12 ^C^
	HT	^a^ 0.03 ± 0 ^D^	^c^ 0.03 ± 0 ^D^	^f^ 1.91 ± 5.72 ^E^
	OT	^b^ 0.04 ± 0.15 ^F^	^d^ 0.14 ± 0.15 ^G^	^g^ 2.01 ± 6.24 ^G^
NH	C	^a^ 0.19 ± 0.04 ^A^	^c^ 0.34 ± 0.08 ^B^	^e^ 0.66 ± 0.13 ^C^
	HT	^ab^ 0.22 ± 0.05 ^D^	^d^ 0.57 ± 0.12 ^E^	^ef^ 0.78 ± 0.24 ^F^
	OT	^b^ 0.24 ± 0.03 ^G^	^d^ 0.49 ± 0.04 ^H^	^f^ 0.88 ± 0.17 ^I^
SN	C	^a^ 8.49 ± 0.25 ^A^	^c^ 15.44 ± 1.54 ^B^	^e^ 18.80 ± 1.15 ^B^
	HT	^b^ 9.15 ± 0.43 ^C^	^d^ 17.71 ± 1.69 ^D^	^f^ 23.33 ± 4.33 ^D^
	OT	^b^ 9.47 ± 0.45 ^E^	^d^ 17.60 ± 1.57 ^F^	^ef^ 20.77 ± 3.34 ^F^

*n* = 10, *p* < 0.05, D1; Day 1, D7; Day 7, D28; Day 28, BF; Bulk-Fill Composite, NH; Nanohybrid Composite, SN; Suprananohybrid Composite, C; No Bleaching Agent Application, HB; Home Type Bleaching Agent Application, OB; Office Type Bleaching Agent Application, Different superscript lower case letters indicate the difference within the same column, and different upper-case letters indicate the difference within the same line.

**Table 6:** Mean values and standard deviations (Mean ± SD) of residual BPA eluted from composites in different periods of time.

		D1	D7	D28
BF	C	^a^ 7.85 ± 1.03 ^A^	^b^ 11.28 ± 1 ^B^	^c^ 19.57 ± 25.12 ^B^
	HT	^a^ 8.5 ± 0.74 ^C^	^b^ 11.96 ± 0.89 ^D^	^d^ 25.00 ± 30.85 ^E^
	OT	^a^ 8.73 ± 0.67 ^F^	^b^ 12.23 ± 0.9 ^G^	^d^ 25.24 ± 31.64 ^H^
NH	C	^a^ 1.12 ± 0.08 ^A^	^c^ 1.45 ± 0.18 ^A^	^e^ 3.23 ± 0.54 ^B^
	HT	^b^ 1.28 ± 0.1 ^C^	^d^ 1.86 ± 0.27 ^D^	^f^ 3.16 ± 0.50 ^E^
	OT	^b^ 1.40 ± 0.19 ^F^	^d^ 1.60 ± 0.3 ^F^	^e^ 3.79 ± 0.55 ^G^
SN	C	^a^ 68.96 ± 3.11 ^A^	^c^ 84.57 ± 6.39 ^B^	^f^ 133.01 ± 17.74 ^C^
	HT	^b^ 76.69 ± 3.07 ^D^	^d^ 97.34 ± 10.57 ^E^	^f^ 136.82 ± 20.14 ^F^
	OT	^b^ 76.01 ± 1.88 ^G^	^e^ 115.83 ± 9.96 ^H^	^f^ 136.06 ± 10.72 ^H^

*n* = 10, *p* < 0.05, D1; Day 1, D7; Day 7, D28; Day 28, BF; Bulk-Fill Composite, NH; Nanohybrid Composite, SN; Suprananohybrid Composite, C; No Bleaching Agent Application, HB; Home Type Bleaching Agent Application, OB; Office Type Bleaching Agent Application, Different superscript lower case letters indicate the difference within the same column, and different upper-case letters indicate the difference within the same line.

**Table 7:** Mean values and standard deviations (Mean ± SD) of residual UDMA eluted from composites in different periods of time.

		D1	D7	D28
BF	C	^a^ 5.08 ± 0.48 ^A^	^b^ 10.42 ± 1.21 ^B^	^c^ 10.71 ± 2.57 ^B^
	HT	^a^ 5.38 ± 0.65 ^C^	^b^ 11.61 ± 1.67 ^D^	^cd^ 12.40 ± 2.61 ^D^
	OT	^a^ 5.52 ± 0.84 ^E^	^b^ 10.92 ± 1.34 ^F^	^d^ 12.9 ± 3.43 ^F^
NH	C	^a^ 42.02 ± 3.03 ^A^	^c^ 68.73 ± 9.4 ^B^	^d^ 79.06 ± 7.06 ^B^
	HT	^b^ 51.60 ± 5.19 ^C^	^c^ 79.68 ± 15.29 ^D^	^d^ 82.79 ± 10.85 ^D^
	OT	^b^ 52.30 ± 3.87 ^E^	^c^ 76.60 ± 11.42 ^F^	^d^ 77.69 ± 8.01 ^F^
SN	C	^a^ 1.67 ± 0.09 ^A^	^d^ 3.89 ± 0.34 ^B^	^f^ 5 ± 0.66 ^B^
	HT	^b^ 2.06 ± 0.08 ^C^	^e^ 4.45 ± 0.53 ^D^	^fg^ 5.71 ± 1.03 ^D^
	OT	^c^ 2.21 ± 0.08 ^E^	^de^ 4.21 ± 0.24 ^F^	^g^ 5.91 ± 0.58 ^G^

*n* = 10, *p* < 0.05, D1; Day 1, D7; Day 7, D28; Day 28, BF; Bulk-Fill Composite, NH; Nanohybrid Composite, SN; Suprananohybrid Composite, C; No Bleaching Agent Application, HB; Home Type Bleaching Agent Application, OB; Office Type Bleaching Agent Application, Different superscript lower case letters indicate the difference within the same column, and different upper-case letters indicate the difference within the same line.

**Table 8:** Mean values and standard deviations (Mean ± SD) of residual Bis-GMA eluted from composites in different periods of time.

		D1	D7	D28
BF	C	^a^ 4.18 ± 0.66 ^A^	^b^ 5.28 ± 0.73 ^B^	^c^ 4.32 ± 0.18 ^A^
	HT	^a^ 4.37 ± 0.51 ^C^	^b^ 5.57 ± 0.82 ^D^	^d^ 5.13 ± 0.37 ^D^
	OT	^a^ 4.7 ± 0.66 ^E^	^b^ 5.37 ± 0.79 ^EF^	^d^ 5.56 ± 0.6 ^F^
NH	C	^a^ 14.90 ± 1.4 ^A^	^d^ 23.37 ± 2.27 ^B^	^f^ 26.09 ± 3.64 ^B^
	HT	^b^ 17.18 ± 1.92 ^C^	^de^ 26.1 ± 2.55 ^D^	^f^ 26.69 ± 4.05 ^D^
	OT	^c^ 19.68 ± 1.99 ^E^	^e^ 28.78 ± 4.94 ^F^	^g^ 33.09 ± 7.28 ^F^
SN	C	^a^ 30.91 ± 1.35 ^A^	^c^ 54.88 ± 3.5 ^B^	^e^ 73.74 ± 7.90 ^C^
	HT	^a^ 35.88 ± 2.61 ^D^	^cd^ 58.00 ± 4.18 ^a E^	^e^ 85.70 ± 14.05 ^F^
	OT	^b^ 37.71 ± 1.38 ^G^	^d^ 61.24 ± 5.37 ^H^	^e^ 80.97 ± 14.65 ^I^

*n* = 10, *p* < 0.05, D1; Day 1, D7; Day 7, D28; Day 28, BF; Bulk-Fill Composite, NH; Nanohybrid Composite, SN; Suprananohybrid Composite, C; No Bleaching Agent Application, HB; Home Type Bleaching Agent Application, OB; Office Type Bleaching Agent Application, Different superscript lower case letters indicate the difference within the same column, and different upper-case letters indicate the difference within the same line.

## Data Availability

Not applicable.
